# Epigenetic Mechanisms of Tamoxifen Resistance in Luminal Breast Cancer

**DOI:** 10.3390/diseases5030016

**Published:** 2017-07-06

**Authors:** Hany A. Abdel-Hafiz

**Affiliations:** Department of Medicine/Endocrinology, School of Medicine, University of Colorado, Ms 8106 PO Box 6511, 12801 E 17th Avenue, Aurora, Denver, CO 80010, USA; Tel.: +1-303-724-1013; Fax: +1-303-724-3920; hany.abdel-hafiz@ucdenver.edu

**Keywords:** epigenetics, DNA methylation, histone modification, microRNA, luminal breast cancer, tamoxifen resistance, cancer stem cells

## Abstract

Breast cancer is one of the most common cancers and the second leading cause of cancer death in the United States. Estrogen receptor (ER)-positive cancer is the most frequent subtype representing more than 70% of breast cancers. These tumors respond to endocrine therapy targeting the ER pathway including selective ER modulators (SERMs), selective ER downregulators (SERDs) and aromatase inhibitors (AIs). However, resistance to endocrine therapy associated with disease progression remains a significant therapeutic challenge. The precise mechanisms of endocrine resistance remain unclear. This is partly due to the complexity of the signaling pathways that influence the estrogen-mediated regulation in breast cancer. Mechanisms include ER modifications, alteration of coregulatory function and modification of growth factor signaling pathways. In this review, we provide an overview of epigenetic mechanisms of tamoxifen resistance in ER-positive luminal breast cancer. We highlight the effect of epigenetic changes on some of the key mechanisms involved in tamoxifen resistance, such as tumor-cell heterogeneity, ER signaling pathway and cancer stem cells (CSCs). It became increasingly recognized that CSCs are playing an important role in driving metastasis and tamoxifen resistance. Understanding the mechanism of tamoxifen resistance will provide insight into the design of novel strategies to overcome the resistance and make further improvements in breast cancer therapeutics.

## 1. Introduction

Breast cancer is one of the most common cancers in women worldwide and is a leading cause of cancer-related death in women in the United States [1]. Based on the expression of estrogen and progesterone receptors (ER/PR) and human epidermal growth factor receptor (HER2) status, breast cancer is classified into further subgroups that include: luminal A (ER+/PR+/HER2 negative), luminal B (ER+/PR+/HER2+), HER2 positive (ER-/PR-/HER2+), basal-like or triple negative (ER-/PR-/HER2−), claudin-low and normal-like [2]. These subgroups are associated with distinct pathological features and clinical outcomes [3]. Clinicians depend mainly on this immunopathological classification in the therapeutic decision-making process [4]. Recently, the American Society of Clinical Oncology/College of American Pathologists (ASCO/CAP) guidelines have defined the absence of ER/PR as less than 1% expression [5]. Approximately 70% of breast cancers express ER and/or PR, followed by triple negative breast cancers (TNBC 19%). The remaining are HER2-overexpressing breast cancer [1]. The presence of ER is considered a good prognostic marker and is commonly used to identify tumors that may respond to endocrine therapy targeting ER signaling pathways. HER2 subtype tumors can be treated by anti-HER2 monoclonal antibodies targeting HER2-dependnet signaling pathway. The TNBC subtype presents the worst prognosis subtype, since it is lacking targeted therapeutic options. Endocrine therapy is the main method of choice to treat luminal breast cancers. However, the development of endocrine resistance represents the main challenge to clinicians. In this review, we discuss recent efforts to understand epigenetic mechanism(s) of endocrine resistance and to resensitize resistant tumors to endocrine therapy.

## 2. Predisposition to Drug Resistance among Luminal Breast Cancer Patients

The luminal subtype tumors respond to ER-targeted therapies such as the mixed antiestrogen tamoxifen and the pure antiestrogen fulvestrant or to estrogen (E) deprivation therapies using aromatase inhibitors such as anastrozole, exemestane and letrozole [6,7]. However, for reasons that are unclear, over 30% of ER-positive (ER^+^) tumors are intrinsically hormone resistant (de novo resistance) at diagnosis, and among hormone-responsive ER^+^ tumors, the clinical behavior can be markedly heterogeneous even when they express similar ER levels [8]. Furthermore, approximately 40% of breast tumors that initially respond to hormone therapies eventually acquire resistance [8].

Luminal breast cancers are subdivided into two molecularly-distinct subtypes, luminal A and B, representing the majority of breast cancers. Genomic efforts have identified many genetic, epigenetic and transcriptional differences between these two luminal subtypes. They respond differently to endocrine therapy, with the luminal A subtype having a better prognosis and being more sensitive to endocrine therapy compared to the luminal B subtype [9]. Patients with luminal A breast cancers respond well to endocrine therapy, and the addition of chemotherapy provides minimal or no clinical benefit to patients with luminal A breast cancer [10]. Gene expression profiling demonstrates that the luminal A subtype is characterized by high ER and PR expression and low expression of proliferation (Ki67 < 14%) and growth factor genes, while the luminal B subtype expresses lower ER, lower or absent PR and high proliferation (Ki67 > 14%) and growth factor receptor gene expression. Likely to have poorer outcomes and to become metastatic [11,12,13,14,15]. In fact, the recurrence rate for luminal B tumors is similar to that of triple negative and HER2 positive tumors [3]. Several studies use different methods to distinguish between these two subtypes including the gene signature that predicts for a specific subtype such as Oncotype DX and MammaPrint [16,17,18]. The genomic signature and biomarkers help to identify groups of patients that benefit from endocrine therapy.

**Figure 1:**
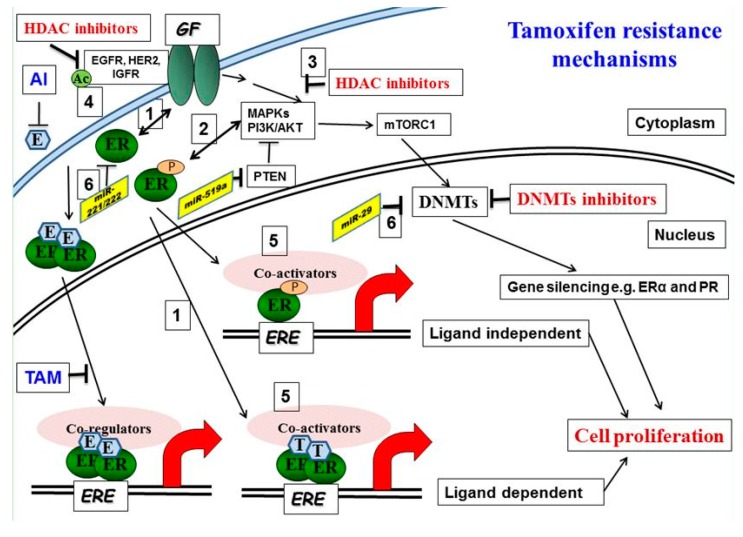
Molecular mechanisms of tamoxifen resistance: (1) Increased bidirectional ER/growth factor (GF) receptor cross-talk converts tamoxifen into an agonist. (2) Activated downstream kinases, including ERK 1, 2 mitogen-activated protein kinase (MAPK) and AKT, phosphorylate both the ER and its accessory proteins. (3) HDAC inhibition reduces phosphorylation of MAPKs and AKT. (4, 5) Acetylation of EGFR promotes receptor tyrosine kinase (RTK) phosphorylation and activation. Corepressor complexes with NCoR Fare inactivated and dismissed from Tam-bound ER-promoter complexes, allowing instead the recruitment of the phosphorylated/activated coactivator complexes with AIB1. This results in an increase in the agonist versus the antagonist activity of tamoxifen on gene transcription. (6) microRNA involved in tamoxifen resistance, miRNA221/222, is upregulated and miR-29 is downregulated in tamoxifen-resistant cells.

Hormone resistance of ER^+^ tumor cells appears to be due to a variety of factors (Figure 1). Examples include expression of mutant ERs or activation of E-independent growth factor signaling pathways. For the latter, activation of epidermal growth factor (EGFR/HER2) or insulin-like growth factor (IGFR) receptor pathways is the major culprit [19]. Unfortunately, these observations have not been translated into effective clinical treatments. Trials combining hormone therapies with EGFR inhibitors have shown little added benefit over hormone treatments alone [20,21], possibly because the appropriate subset of tumors likely to benefit have not been identified. Thus, tumors that relapse under continued hormone therapies remain an elusive problem. In order for long-term tumor suppression to be achieved, hormone responsiveness restoration remains an important clinical priority.

### The Molecular Mechanisms behind Luminal Tumor-Cell Heterogeneity

Cancer cells within solid tumors, especially in luminal breast disease, exhibit striking heterogeneity characterized by multiple phenotypic and genotypic cell subpopulations [22,23]. It is important to define these cell subpopulations to develop rational methods for targeting them and understand how each contributes to hormone resistance [24]. Non-genetic hypotheses speculate that some cell subpopulations are paradoxically destined for clonal expansion in response to stimuli like antiestrogens. Other hypotheses suggest that epigenetic, environmental or even dietary factors play a role in resistance. Tumor cell heterogeneity has been assessed with genetic changes such as copy number alteration and somatic mutation. However, recent studies have shown that epigenetic heterogeneity leads to cell-to-cell variation in response to therapy [25]. These epigenetic mechanisms include DNA methylation, histone post-translational modifications and chromatin remodeling (as discussed in next section). Epigenetic alterations are linked to genetic mutation of epigenome regulators, such as the writers, readers and erasers of epigenetic markers [25].

It has also been suggested that resistant cells emerge from a subpopulation of cancer-initiating/cancer stem-like cells, which gain growth advantages via epigenetic mechanisms. Thus, the origins of luminal breast cancer-cell heterogeneity remain unclear in regards to the roles of stem cells, somatic mutations, copy number changes and epigenetic alterations of genes involved in tumor growth, invasion, metastasis and resistance. Luminal tumor-cell heterogeneity is a continuing issue, for example the current clinical classifications that define luminal breast cancers as ones having at least 1% ER^+^ or PR^+^ cells [26]. This percentage of heterogeneity immediately raises practical questions: If so, what are the other 99% of cells? How do they contribute to tumor aggressiveness? How do they contribute to the development of hormone resistance? What treatment strategies could possibly be designed for such complex tumors?

## 3. Epigenetic Regulation of Gene Signaling in Breast Cancers

Epigenetic modifications are inheritable changes in gene expression without alterations in the DNA sequence. Compared to genetic changes, epigenetic modifications are often enzymatic and can be reversed by epigenetic inhibitors. In the nucleus, double-stranded DNA is compacted and organized into chromosomes. The DNA is wrapped around histone protein-complexes to form larger order nucleosomal structures, the basic structural units of chromosomes, allowing the selective accessibility of transcription machinery. The degree of DNA coiling determines whether chromatin is “open, euchromatin” and available for transcription or “closed, hetero-chromatin” and transcriptionally repressed. The balance between euchromatin and hetero-chromatin is determined by epigenetic regulation, allowing cells to regulate gene expression, resulting in significant changes in their biological functions. Epigenetic modifications include methylation of DNA, modification of histone proteins and alteration of miRNA expression; all of which influence gene expression patterns [27,28]. Since epigenetic modifications can be reversed, they appear to be desirable therapeutic targets for cancer patients. Below, we discuss each of these in detail.

### 3.1. DNA Methylation

DNA methylation is the most important epigenetic modification in mammalian cells that is associated with gene expression. DNA methylation is associated with normal development and growth [29] and is dysregulated in tumors [30]. The covalent addition of methyl residues to cytosines residing in CpG dinucleotides is catalyzed by DNA methyltransferases (DNMTs) including DNMT1, DNMT3a and DNMT3b [29]. DNMT1 is required for maintenance of established DNA methylation patterns. Its deficiency may lead to global hypomethylation [31,32]. DNMT3a and DNMT3b are implicated in the generation of de novo methylation patterns [33]. Additionally, a family of nuclear methyl-CpG-binding protein domain (MBD) binds to methylated cytosines on DNA and regulates gene expression [34,35]. MBDs regulate gene transcription by recruiting histone modifying complexes such as the nucleosome remodeling deacetylase NuRD complex and histone methyltransferases (HMTases) [36]. In humans, ~70% of all CpG islands are hypermethylated and located in tightly-packed core regions of DNA. In contrast, CpG islands that remain hypomethylated are found in relaxed, open, frequent promoter regions of DNA, which enables gene expression [37].

It is estimated that 30% of breast cancer is linked to epigenetic modifications, particularly in DNA methylation [38]. Changes in DNA methylation patterns have been shown to be associated with breast cancer development, progression and metastasis [39]. Breast cancer has been associated with hypermethylation of tumor suppressor genes and with hypomethylation of oncogenes [38]. For example, the number of genes has been reported to be methylated and consequently silenced, including tumor suppressor genes, such as secreted frizzled-related protein (*SFRP*), *RASSF1A,* WNT inhibitory factory factor 1 (*WIF1*) inter-alpha-trypsin inhibitor heavy chain family member 5 (ITIH5), Dickkopf WNT signaling pathway inhibitor 3 (*DKK3*), ATM serine/threonine kinase (*ATM*), long interspersed nuclear element 1 (*LINE1*), cyclin-dependent kinase inhibitors (*CDKN2* and *CDKN1B*), G1/S-specific cyclin-D2 (*CCND2*), breast cancer 1 ( *BRCA1*), mutL homology 1 (*MLH1*), glutathione S-transferase P (*GSTP1*), homeobox protein (*HOXA5*), cadherin-1 (*CDH1*), metalloproteinase inhibitor 3 (*TIMP3*), cAMP-responsive element-binding protein 3-like protein 1 (*CREB3L1*) and hormone receptors (*ESR1* and *PGR*). These genes have been shown to be involved in cell cycle regulation, DNA repair, cell detoxification, apoptosis and cell adhesion and invasion [38,40,41,42,43,44,45,46,47]. The phospholipase A2 receptor (PLA2R1) is a transmembrane protein that plays a role in the clearance of phospholipase A2. The phospholipase A2 receptor (PLA2R1) acts as a tumor suppressor in certain tumors including breast cancer. PLA2R1 has been shown to be differentially expressed in normal and mammary cancer cells, and this expression is controlled by epigenetic mechanisms such as DNA methylation and histone modification [48].

DNA methylation signatures have been identified for the characterization and molecular subtyping of breast cancers [49,50,51,52,53,54]. Holm et al. report that Luminal B tumors has been shown to be more methylated than Basal-like or triple negative breast cancers and may contribute to tumor progression in this subtype [55,56]. In general, DNA methylation plays an important role in different subtypes of breast cancers, thereby providing valuable information on disease prognosis and response to treatment. Inhibition of DNMTs by cytosine nucleoside analogs such as 5-Aza-2′-deoxycytidine (AZA; decitabine) has been widely used to investigate the role of DNA methylation in breast cancer.

### 3.2. Histone Modifications

Post-translational modifications (PTMs) of histone tails such as phosphorylation, ubiquitination, SUMOylation, acetylation and methylation play an important role in modifying gene expression [57,58]. These modifications change the secondary structure of DNA and result in either induction or prevention of access by transcription factors to gene promoter regions.

Histone acetylation is a dynamic reaction catalyzed by histone acetyltransferases (HATs) or histone deacetylases (HDACs). HATs acetylate ε-amino groups of lysine residues in the N-terminal tails of core histones, relaxing chromatin and allowing transcription factor binding. The acetyl groups are removed from lysines by histone deacetylases (HDACs), which compact chromatin into tightly ordered nucleosomes and prevents access of transcription factors to DNA. In general, transcription activators recruit coactivators such as p300/CBP with HAT activity to DNA sites, whereas transcription repressors recruit corepressors with HDAC activity [59]. Histones can also be methylated, which turns genes “off", or demethylated, which turns them “on”, by loosening or either tightening histone tails, which allows or restricts transcription factor loading on DNA [58,60].

#### 3.2.1. Histone Acetylation and HAT Inhibitors

HATs can stimulate or suppress tumor growth and progression. Depending on the target gene, hyper-acetylation of oncogenes leads to cancer progression. For example, increased histone acetylation was detected in hepatocellular carcinoma and is associated with prostate cancer recurrence [61]. The human HATs are classified into two types; type A HATs are nuclear enzymes responsible for acetylation of histones and non-histone proteins in the nucleolus, while type B HATs are cytoplasmic enzymes that modify free histones in the cytoplasm and then transport to the nucleus. Based on sequence homology, HATs are divided into three families: the GNAT (GCN5-related *N*-acetyltransferases) family consists of KAT2A and KAT2B; the MYST family (MOZ, Ybf2/Sas3, Sas2 and Tip60); and orphan HATs that include p300/CBP and steroid receptor coactivators (SRCs) [62,63,64]. The HAT enzymes have various substrate specificities for histone and non-histone proteins [61]. Because of their involvement in cancer development, HATs were proposed to be promising targets. Histone acetyltransferase MYST3 plays an important role in breast cancer development and activation of ER expression, and targeting MYST3 may serve as a novel strategy to block ER expression in MYST3-high ER+/HER2− breast tumors [65].

HAT modulators suppress the catalytic activity of the acetyl transferases. However, only a very limited amount of HAT modulators has been described or investigated [66], which are classified into bisulfate inhibitors, natural products, synthetic analogues and derivatives and small molecules (reviewed in [61,62,67]). These modulators have been limited to in vitro studies of growth inhibition of cancer cells [68]. Several small molecule HAT inhibitors have been derived from natural products, such as garcinol, curcumin and anacardic acid [61]. The lack of cellular permeability represents a major challenge of some of HAT inhibitors [69]. For example, anacardic acid, isolated from the shells of cashew nuts, is a potent in vitro inhibitor of both p300 and PCAF’s HAT activity [70]. Because cells are poorly permeable to anacardic acid, synthetic analogs are being analyzed for their HAT-inhibitory activity and effects on cancer cells [71].

#### 3.2.2. Histone Deacetylation and HDAC Inhibitor

HDACs fall into two classes based on their structure: zinc-dependent classes I, II and IV; and NAD-dependent class III, also called sirtuins [72]. Class I consists of HDACs 1, 2, 3 and 8, whereas class IV has only one member HDAC11. Class II is divided into class IIa (HDAC 4, 5, 7, 9) and class IIb (HDAC 6 and 10) [73]. HDACs have been shown to have an important role in cancer development and progression. Previous reports indicate that HDAC levels are increased in certain types of cancer [74]. For example, HDAC1 is expressed in many cancers such as prostate, gastric, esophageal and breast cancers (reviewed in [74]). HDAC inhibitors increase cellular protein acetylation by inhibiting HDAC activity. There are four classes of HDAC inhibitors: hydroxamic acids (suberoylanilide hydroxamic acid (SAHA, vorinostat)), benzamides (MS-275), cyclic peptides (romidepsin) and short-chain fatty acids (valproic acid) [75]. HDAC inhibitors have been shown to inhibit tumor growth and promote apoptosis of cancer cells, while not affecting normal tissue [76,77]. Several clinical trials using HDAC inhibitors have been performed, and the results indicate that HDAC inhibitors have anticancer activity [74]. For example, HDAC inhibitors SAHA and romidepsin (FK228) were approved by the U.S. Food and Drug Administration (FDA) for the treatment of cutaneous T-cell lymphoma [78]. In addition, vorinostat, entinostat and panobinostat (LBH-589) have been demonstrated to exhibit potent activity when combined with cytotoxic drugs (paclitaxel), endocrine (tamoxifen) therapies; or with therapies targeted at HER2 (trastuzumab) or VEGF (bevacizumab) [41,45,74,79]. It has been shown recently that the combination of the HDAC inhibitor YCW1 with ionizing radiation induce cell death in triple-negative breast cancer cell lines in vivo and in mouse models [80].

#### 3.2.3. Histone Methylation and Demethylation

Histone methylation occurs on the side chains of lysine (K) and arginine (R) residues; however, unlike acetylation, there is no change in the charge of the histone protein. Histone methylation is regulated by histone methyltransferases (HMTs) and histone demethylases (HDMs) [81]. Histone lysine methylation is associated with both transcriptional activation and repression. Methylation of histone 3 lysine 9, 20 or 27 (H3K9, H3K20 or H3K27) is associated with transcription silencing, whereas methylation of histone 3 lysine 4, 36 or 79 (H3K4, H3K36 or H3K79) is associated with transcription activation [57]. Beside gene transcription, histone methylation markers also recruit proteins associated with DNA repair [81]. Histone methylation regulates many cellular functions, including gene transcription, DNA replication and repair, developmental and differentiation processes, pluripotency and maintenance of genome integrity. It also affects the development of many diseases including malignancies [82]. Targeting histone methylation enzymes may restore normal methylation profile. Histone methylation is catalyzed by three families of enzymes, the set-domain containing protein family, the non-set domain protein family and the PRMT1 (protein arginine methyltransferases) family [83].

Enhancer of zeste homolog 2 (EZH2), a polycomb repressive complex 2 (PRC2) group protein, is a histone methyltransferase that methylates H3K27 and functions as a transcriptional repressor [84]. The overexpression of EZH2 is strongly associated with the development of breast cancer and breast cancer’s aggressiveness [85,86,87]. Indeed, it has been shown that EZH2 inhibits the expression of several tumor suppressor genes such as P16 INK4a, E-cadherin, BRCA1 and the adrenergic receptor β2 [82]. 3-Deazaneplanocin (DZNep), an inhibitor of the EZ2H, was widely used for experimental work. In spite of promising results, DZNep has a short plasma half-life, has nonspecific inhibition of histone methylation and is toxic in animal models [88]. DZNep induces antitumor activity and apoptosis in breast cancer cells, but not in normal cells [89]. Several EZH2 inhibitors have been developed in order to improve antitumor activity and reduce toxicity (reviewed in [88]). For example, tanshindiols are small molecule inhibitors of EZH2 that also possess anti-cancer activity in several tumor cell lines [90]. Lastly, inhibitory EZH2 peptides have been designed among which one termed SQ037 has been validated and shown to have considerable anti-EZH2 potency [91]. Some of these inhibitors have been moved to clinical trials, show early promising results and would be expected to have the desired efficacy with minimal side-effects [91].

The set and MYND domain containing protein 3 (SMYD3) is a novel histone lysine methyltransferase, and it specifically methylates H3K4 (reviewed in [92]). SMYD3 plays a significant role in the development and progression of human cancer via regulating gene transcription and promoting cells proliferation and migration. SMYD3 is overexpressed in several malignancies including esophageal squamous cell carcinoma, gastric cancer, hepatocellular carcinoma, prostate cancer, leukemia and breast cancers. Silencing using small interfering RNAs inhibits the growth of these cancer cells [93]. Similarly, inhibition of SMYD3 expression by Novobiocin inhibits the proliferation and migration of MDA-MB-231 breast cancer cells in a dose-dependent manner. The effect of Novobiocin is associated with the downregulation of SMYD3. Tranylcypromine is another potent H3K4 methylase. This small molecule demethylation inhibitor de-represses transcription of important target genes including the pluripotent stem cell marker Oct4 [94,95].

Lysine-specific demethylase 1 (LSD1) removes methyl groups from methylated proteins including histone H3 (H3K4) and non-histone proteins such as p53 and DNMT1, suggesting that it is involved in a wide variety of normal biological functions [96,97,98,99,100]. LSD1 is overexpressed in various types of solid tumors including basal-like breast cancer where it is a biomarker of poor prognosis and aggressiveness [101,102]. It has been shown recently that LSD1 is a potential target gene of miR-708. Overexpression of miR-708 inhibits breast cancer cell line MDA-MB-231 proliferation and invasion, whereas inhibition of miR-708 enhances these processes [103]. LSD1 together with ER remove methyl groups from H3K9 to activate gene expression [104]. There are several inhibitors of LSD1 that have been developed and tested for their effects on many forms of cancers. These include bizine, the tranylcypromine derivatives NCL1 and GSK2879552, biguanide and bisguanidine polyamine analogs [105]. GSK287 is an orally-bioavailable irreversible LSD1 inhibitor, currently under clinical evaluation for cancer treatment [106]. These LSD1 inhibitors alter promoter activity of multiple genes in breast cancer cells and are postulated to have considerable therapeutic potential [107,108,109]. It has been shown that LSD1 interacts with HDACs to control breast cancer cell growth. Combined treatment of triple-negative breast cancer cells with LSD1 inhibitor, pargyline, and HDAC inhibitor, SAHA, leads to growth inhibition [110,111].

G9a, a histone methyltransferase responsible for H3K9 methylation, has been reported to promote cancer aggressiveness, and its overexpression has been associated with poor prognosis [112,113,114,115,116]. Several small molecules inhibitors have been developed to inhibit the enzymatic activity of G9a [116]. Inhibition of G9a reduces the invasiveness and metastatic potential of human lung cancer cells [117] and inhibits the growth of prostate cancer cells [118]. BIX01294 (a diazepine-quinazoline-amine derivative) is one of the first molecules developed to reduce G9a-mediated H3K9 di-methylation [119]. BIX01294 treatment was shown to inhibit proliferation, motility and invasiveness of human neuroblastoma cells [120] and pancreatic cancer cells [121]. UNC0638 is another inhibitor characterized by high potency and specificity for G9a. In vitro, UNC0638 treatment has been shown to inhibit cell proliferation in various cell lines such as breast, squamous head and neck carcinoma, hepatocellular carcinoma, acute leukemia and cervical cancer [116].

### 3.3. microRNAs 

Just as methylation modifies DNA and its ability to be transcribed, it modifies miRNAs and their ability to regulate protein expression post-transcriptionally. Epigenetic hypomethylation on miRNAs that regulate ER signaling is associated with deregulated ER function in breast cancers [122].

miRNAs are short, naturally-occurring noncoding RNAs (18–22 nucleotides in length) that regulate gene expression of target genes involved in different cellular functions including proliferation, differentiation and apoptosis [123,124]. Evidence indicates that alteration of miRNA expression is associated with cancer development and progression [125,126,127,128,129]. Several investigators have defined miRNA signatures that are differentially expressed in breast cancers compared to normal mammary tissues and are able to distinguish between different breast cancer subtypes [128,130,131,132,133]. At normal levels, miRNAs act as tumor suppressors or oncogenes. For example, miRNA-10b, miRNA-125b and miRNA-145, are downregulated, while miRNA-21 and miRNA-155 are upregulated in tumors compared to normal tissues [128]. The putative targets of miRNA-125b are the oncogenes YES, ETS1, TEL and AKT3; the growth factor FGFR2; and members of the MAPK pathway (MAP3K10, MAP3K11 and MAPK14) [128]. On the other hand, upregulated miRNA-21 targets the tumor suppressors PDCD4 and PTEN [127,134]. It has been shown that miRNA-30 expression is correlated with ER and PR levels and that miRNA let-7 isoforms regulate PR status (let-7c), lymph node metastasis (let-7f-1, let-7a-3, let-7a-2) and proliferation indices (let-7c, let-7d). miRNA let-7 also appears to be a tumor suppressor that is downregulated in breast cancer stem cells (CSC) [135,136]. Preclinical studies have shown that miRNAs play a functional role in different steps of the metastatic cascade (reviewed in [137]). Several miRNAs have been shown to act as EMT-negative regulators by targeting specific EMT-associated transcription factors (miR-1, miR-15b, miR-30c, miR-34a, miR-101, miR-124, miR-132, miR-137, miR-138, miR-150, 153, miR-200s, **203,** miR-204, miR-205, miR-300, miR-335). Some miRNAs were found to regulate EZH2 (miR-15b, miR-138) or the NAD-dependent deacetylase sirtuin-1 (miR-200s, miR-204). In addition, miR-451 has been shown to play a role in tamoxifen resistance [82,83].

miRNAs have been shown to be easily extracted from different body fluids including blood, saliva and sputum. Several studies demonstrated that circulating miRNA can be used as biomarkers to discriminate between normal and diseased patients in many cancers, including breast cancer (reviewed in [138]). Overall, the ability of miRNA to very specifically target a desired miRNA has great promise as highly specific targeted therapies for cancer treatment.

## 4. Epigenetic Modulation of Tamoxifen Resistance

### Estrogen Receptor 

The interplay between epigenetics and ER signaling is believed to be one important factor that dictates breast cancer development and tumor response to conventional therapies. ER signaling plays a role in histone modifications including acetylation, phosphorylation and methylation through the interaction with histone modifying enzymes [139]. On the other hand, epigenetic pathways regulate ER signaling by controlling ER expression levels. Moreover, ER target genes are regulated by co-recruitment of ER and epigenetic cofactors, including HATs, HDACs, HMTs, DNMTs and polycomb proteins (reviewed in [140]). The overall balance among these coregulatory proteins controls ER functions including their responses to endocrine therapies. Downregulation of corepressors and/or overexpression of coactivators are very likely to contribute to endocrine resistance. Overexpression of ER co-regulators such as SRC1, TIF2, SRC3 and CBP contributes to tamoxifen resistance [141,142,143]. SRC1 overexpression is associated with tamoxifen resistance and disease recurrence only in the HER2− positive breast cancer subtype [144]. SRC3 overexpression is associated with shorter survival among tamoxifen-treated patients, suggesting that tamoxifen activity may be switched from antagonist to agonist. Cellular distribution of SRC3 has been shown to influence tamoxifen responsiveness. Nuclear SRC3 was associated with a favorable outcome in patients receiving endocrine therapy [145].

Tamoxifen inhibits cancer growth by inhibiting ER transcriptional activity. Its activity depends on the ability to stabilize the binding of ER to corepressors such as NCOR1 and smart (reviewed in [146]). Downregulation of these corepressors has been implicated in endocrine resistance. Girault et al. have shown that weak expression of NCOR1 is significantly associated with shorter relapse-free survival, suggesting that NCOR1 is required for full tamoxifen efficacy [143].

Recruitment of HDACs to an ER-corepressor complex enhances the actions of tamoxifen, while the absence of HDAC recruitment to corepressors at a tamoxifen-bound ER complex results in drug resistance [147]. Inactivation of the ER corepressor could predispose cancer cells to the anti-tumorigenic effect of HDACi, while genomic alterations in ER corepressors or coactivators are candidate biomarkers that could predict response to HDACi in tamoxifen-resistant breast cancers [147]. Along those lines, it has been reported that depletion of the nuclear receptor coactivator SRC3 enhances the sensitivity of breast cancer cells to the HDACi vorinostat (SAHA). In contrast, overexpression of SRC3 decreases SAHA-induced cancer cell apoptosis [148].

## 5. Epigenetic Regulation of Breast Cancer Stem Cells

Breast CSCs are theoretically a rare, immortal cell subset within the heterogeneous population of solid-tumor cancer cells. They can both self-renew and give rise to all other cell subpopulations present in that tumor. Such CSCs would represent a significant clinical challenge as they would not only be resistant to therapies, but would also play essential roles in tumor recurrence and metastasis [149].

Cellular heterogeneity within breast cancers and the putative existence of breast CSCs are also possible reasons for therapeutic failures [150]. Assuming they exist, breast CSCs are predicted to be a minor (possibly <1%) non-proliferative precursor cell subpopulation within a tumor, able to give rise to and maintain more differentiated downstream cell types within a tumor [151,152,153], but are resistant to endocrine, radiation and chemotherapies that target the more differentiated cells [154,155,156,157,158,159,160,161,162,163,164,165]. If so, therapeutic approaches that kill CSCs may be the best hope for curing cancers. However, with regard to human breast CSCs, much uncertainty remains about their identity, markers that identify them and whether there is a single definable breast CSC or if there are multiple CSCs that differ among breast cancer subtypes.

Conventional treatments only kill differentiated cancer cells. Targeting putative CSCs is a promising therapeutic approach, although, especially in luminal breast cancers, identification of these cells remains elusive. One approach would be to target their self-renewal capacity by inducing their differentiation; a switch that would presumably reduce their resistance to drugs. This is not outside the realm of possibility; it has been reported that histone modifier (Bmi-1 and EZH2) and non-coding RNA (let7, miR-93, miR-100 and HOTAIR) are involved in the regulation of CSC phenotypes. For instance, exposure to vorinostat reduces mammosphere formation capacity, an index of CSC function [149]. Witt et al. [166] have shown that when compared to non-stem-tumor-cells, the deacetylases HDAC1 and HDAC7 are overexpressed in CSCs. They demonstrated that currently available HDACi such as trichostatin A, a pan HDACi, suppress HDAC1 and HDAC, and may therefore modify the epigenetic marks that characterize CSCs.

Epigenetic modifications are involved in the formation, maintenance and function of breast CSCs. However, there are not many data on DNA methylation, and its interplay with breast CSCs. DNA methylation has been shown to regulate hematopoietic stem cells where it targets pluripotency factors [152,167,168,169,170,171] and targets CD133 in colon, ovarian, blood, prostate and brain CSCs [172,173,174,175]. The association between DNA methylation and CSCs [176] suggests that hypomethylating agents have the potential to induce CSC differentiation generating cells that would be sensitive to therapeutic agents. A methyltransferase inhibitor, 3-deazaneplanocin, can disrupt the polycomb 2 complex and reduce CSCs in acute myeloid leukemia [177], hepatocellular carcinoma [178], glioblastoma [179] and prostate cancer [180].

In addition to targeting regulatory genes in differentiated cells, histone modifications also play a role in CSC biology. The polycomb repressive complex (PRC), which represses gene expression through histone modification and chromatin compaction [181], regulates CSCs of the breast [182], prostate [180], ovary [183] and glioblastomas [184]. Paranjape et al. have shown that overexpression of Bmil, a polycomb protein, increased self-renewal and stemness in mammary epithelial cells [185]. Inhibitor of *LSD1*, the enzyme responsible for demethylating H3K4, acts specifically on embryonal carcinoma stem cells of pluripotent cancers such as teratocarcinomas, seminomas and embryonic carcinomas [186], suggesting that H3K4 demethylation is involved in the formation of such tumors.

Duru and coworkers [187] have discussed the role of epigenetics in the regulation of miRNA with relation to CSCs. They have shown that miR-140 can be activated by epigenetic therapy or dietary compounds targeting stem cells in ductal carcinoma in situ, thereby preventing relapse or progression to invasive disease.

Histone acetylation also plays an important role in the regulation of CSC miRNAs. For example, miR-34a is downregulated in pancreatic CSCs. HDAC inhibitor SAHA (vorinostat) treatment restores miR-34a levels and decreases CSCs viability. In breast cancers, miR-34a suppresses HDAC1 and HDAC7 expression and its level is inversely correlated with HDAC1 and HDAC7 activity, as well as tumor characteristics such as grade and stage [188]. Therapy-resistant and aggressive breast cancers are associated with low miR-34a expression and high HDAC1 and HDAC7 expression, which deacetylate HSP70K 246 [189].

Previous studies have identified unique miRNA expression profiles for breast CSCs compared to non-tumorigenic cells, and the dysregulation of miRNA plays an important role in breast CSC biology [135,190,191,192,193]. These miRNAs may function as oncomirs or tumor suppressor genes to regulate self-renewal, invasiveness and drug resistance of CSCs [191]. miRNA-200 isoforms a, b and c are downregulated in breast CSCs whose targets include stem cell self-renewal factor Bmi, and the transcriptional repressors of E-cadherin, ZEB1/ZEB2 [192,194]. miRNA let-7, miRNA-200c and miRNA-107 have been shown to be downregulated by Lin28, an RNA-binding protein that induces specific miRNA uridylation and blocks miRNA processing by Dicer [195]. miRNA-103/107 overexpression induces epithelial-mesenchymal transition (EMT) and increases the risk of metastases in breast cancer patients [196]. EMT has been directly linked to the generation of cells with CSC-like properties [197]. Other miRNAs reportedly downregulated in breast CSCs include miRNA-30, miRNA-128, miRNA-34c, miRNA-34a and miRNA-16; while upregulated miRNAs include miRNA-181 and miRNA-495 [190].

## 6. Summary and Future Perspectives

In summary, resistance to hormone therapies in breast cancers can arise from a variety of mechanisms among which epigenetic changes are likely to be important. DNA methylation for instance alters mRNA expression of genes important for estrogen-dependent growth. It is not surprising then that hormone resistance is likely due to a combination of factors that include both a selection of pre-existing resistant cells harboring irreversible genetic defects as well as cells carrying reversible epigenetic errors. The latter is targetable. For this reason, understanding specifically how epigenetic changes contribute to the broad phenotype of hormone resistance could uncover treatment modalities and pathways for which drugs are already available. These could be used either to prevent development of resistance or to restore drug sensitivity to previously resistant cells. However, it is diagnostically important to identify the tumor and cell types that would respond to such treatments. For example, luminal breast cancers respond differently to epigenetic drugs than do basal and receptor-negative breast cancers [198]. Additionally, it is critically important to evaluate the combinatorial effects of hormone therapies together with epigenetic therapies in order to answer even the most rudimentary questions: i.e., should such treatments be combined or sequenced? Despite the large number of studies dealing with epigenetics and breast cancers published in the past few years, much remains to be learned at the basic research level before translational applications can be rationally deployed.
